# Insulin Gene Expression Is Regulated by DNA Methylation

**DOI:** 10.1371/journal.pone.0006953

**Published:** 2009-09-09

**Authors:** Akio Kuroda, Tibor A. Rauch, Ivan Todorov, Hsun Teresa Ku, Ismail H. Al-Abdullah, Fouad Kandeel, Yoko Mullen, Gerd P. Pfeifer, Kevin Ferreri

**Affiliations:** 1 Department of Diabetes, Endocrinology, & Metabolism, Research Institute of City of Hope, Duarte, California, United States of America; 2 Department of Biology, Beckman Research Institute of City of Hope, Duarte, California, United States of America; University of Bremen, Germany

## Abstract

**Background:**

Insulin is a critical component of metabolic control, and as such, insulin gene expression has been the focus of extensive study. DNA sequences that regulate transcription of the insulin gene and the majority of regulatory factors have already been identified. However, only recently have other components of insulin gene expression been investigated, and in this study we examine the role of DNA methylation in the regulation of mouse and human insulin gene expression.

**Methodology/Principal Findings:**

Genomic DNA samples from several tissues were bisulfite-treated and sequenced which revealed that cytosine-guanosine dinucleotide (CpG) sites in both the mouse *Ins2* and human *INS* promoters are uniquely demethylated in insulin-producing pancreatic beta cells. Methylation of these CpG sites suppressed insulin promoter-driven reporter gene activity by almost 90% and specific methylation of the CpG site in the cAMP responsive element (CRE) in the promoter alone suppressed insulin promoter activity by 50%. Methylation did not directly inhibit factor binding to the CRE *in vitro*, but inhibited ATF2 and CREB binding *in vivo* and conversely increased the binding of methyl CpG binding protein 2 (MeCP2). Examination of the *Ins2* gene in mouse embryonic stem cell cultures revealed that it is fully methylated and becomes demethylated as the cells differentiate into insulin-expressing cells *in vitro*.

**Conclusions/Significance:**

Our findings suggest that insulin promoter CpG demethylation may play a crucial role in beta cell maturation and tissue-specific insulin gene expression.

## Introduction

Insulin is a master regulator of metabolic homeostasis and it is secreted from pancreatic beta cells in response to nutrient stimulation. Insulin gene expression begins early in the embryonic development of the pancreas and remains tightly-regulated throughout adult life. The mouse genome contains two insulin genes, *Ins1* (GeneID: 16333) and *Ins2* (GeneID: 16334). The *Ins2* gene has greater structural and functional similarity to other mammalian insulin genes, whereas *Ins1* is considered to be a functional retroposon since it possesses only one of the two introns present in *Ins2* and other mammalian insulin genes [1]. Mouse *Ins2*, but not *Ins1*, is expressed in the thymus [2], yolk sac [3], fetal liver [4], and brain [5] as well as the pancreas, similar to the human gene. Additionally, both *Ins2* and the human *INS* (GeneID: 3630) gene, but not *Ins1*, are paternally imprinted in the yolk sac [6] in association with the H19/Igf2 imprint control region [7], [8], but exhibit biallelic expression in the adult pancreas [9]. Since imprinting of this region is associated with differential DNA methylation patterns [10], [11], we hypothesized that insulin gene expression may be regulated by DNA methylation.

Mammalian gene promoters contain cytosine-guanosine dinucleotide (CpG) sites which play a pivotal role in the control of gene expression [12], [13]. Methylation of the cytosine residues regulates transcription directly by inhibiting the binding of specific transcription factors, and indirectly by recruiting methyl-CpG-binding proteins and their associated repressive chromatin-remodeling activities [14]. These epigenetic alterations are responsible for modulation of developmentally-regulated and tissue-specific gene expression [15]. DNA sequences that regulate expression of insulin gene are located within approximately 400 nucleotides upstream of the transcription start site [16] and direct both tissue-specific and metabolic-responsive transcription of the insulin gene. Adjacent to the promoter is a microsatellite variable number tandem repeat (VNTR) region which is also important for insulin gene expression and variability in this region is associated with both type 1 (class I VNTR) and type 2 (class III VNTR) diabetes mellitus [17]. The pathophysiology of type 1 diabetes (T1DM) is the result of the destruction of the insulin-producing beta cells, and a promising treatment paradigm for T1DM is replacement of the missing beta cells with insulin-producing cells derived from embryonic or other stem cell sources. Many groups worldwide are currently working to develop methods to produce beta cells in vitro, however as yet there are no reports demonstrating fully functional cells appropriate for cell replacement therapy. A major hurdle has been achieving normal levels of insulin gene expression.

Although much is known about the control of insulin gene expression, DNA methylation of the insulin promoter and its relationship to gene expression has not been investigated. To understand the role of epigenetic regulation in beta cells, we examined the DNA methylation pattern of the insulin gene in human and mouse beta and non-beta cells and in embryonic stem cells as they differentiate into insulin-expressing cells.

## Results

### Beta cell-specific demethylation of the insulin gene

**Figure 1:**
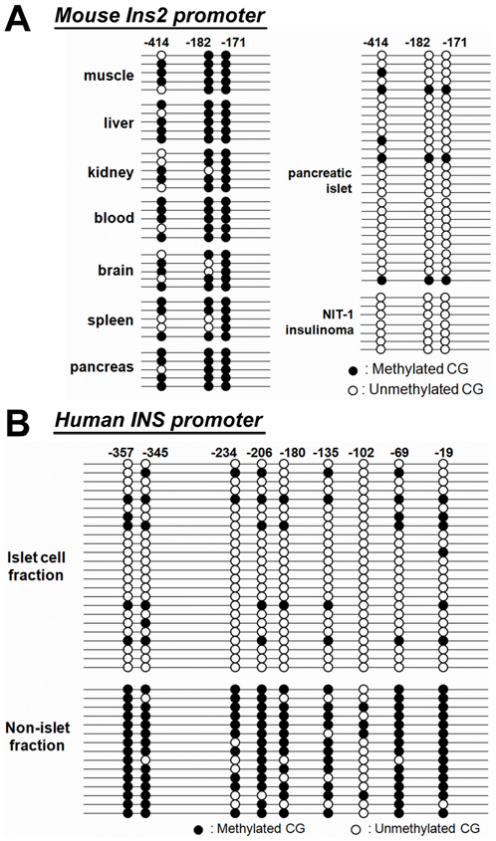
Tissue-specific methylation of the mouse *Ins2* and human *INS* promoters. (A) Genomic DNA samples from mouse muscle, liver, kidney, blood, brain, spleen, whole pancreas, partially purified pancreatic beta cells (∼70%), and the mouse NIT-1 beta cell line were analyzed for tissue specific methylation patterns of the mouse *Ins2* promoter (position −480 to +15 bp) which contains three potential methylation sites. (B) Genomic DNA samples obtained from human beta cells (“Islet cell fraction”; ∼70% islet cells) and from human pancreatic exocrine tissue (“Non-islet fraction”; <1% islet cells) were analyzed for methylation of the insulin promoter (−385 – +24 bp), which contains nine CpG sites. Note that 7 of 24 clones (29%) of the clones in the beta cell population exhibited some CpG methylation, consistent with the percentage of non-beta cell contamination.

**Figure 2:**
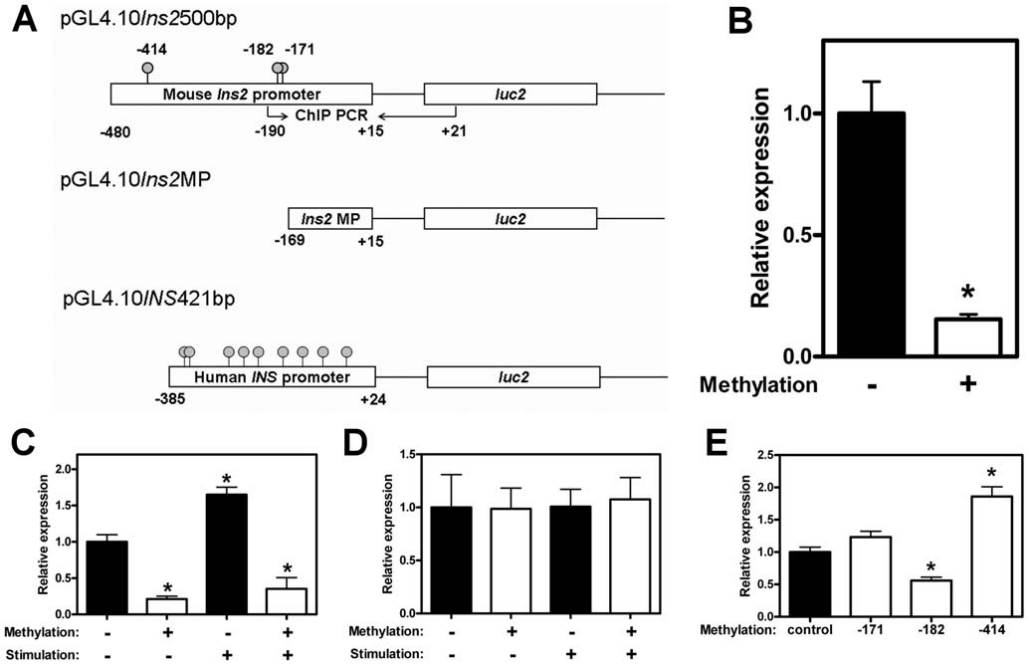
Methylation suppresses the activity of both the mouse and human insulin gene promoters. (A) A diagram of the three luciferase reporter plasmids used to test the effect of methylation on insulin promoter activity. PGL4.10*Ins2*500bp contains bases −480 to +15 (three CpG sites) of the *Ins2* gene, pGL4.10*Ins2*MP contains bases −169– +15 bp (no CpG sites), and, pGL4.10*INS*421bp contains bases −385 to +24 of the human *INS* gene (nine CpG sites). The positions of CpG dinucleotides are represented with “lollipop” markers and the positions of primers for the ChIP experiments by arrows. (B) Methylated (white bar) or mock-methylated (black bar) human *INS* promoter constructs were transfected into NIT-1 insulinoma cells 48 hours prior to the luciferase assay. The data were normalized with co-transfected control vector and are the average and standard error from three separate experiments of eight replicates each. Data are presented as relative expression compared with the non-methylated, non-stimulated sample. * = p<0.0001 (C) Methylated (white bars) or mock-methylated (black bars) mouse *Ins2* promoter constructs were transfected into NIT-1 insulinoma cells and stimulated for four hours with 2.8 mM glucose (Stimulation -) or with 16.7 mM glucose and 10 µM forskolin (Stimulation +). The data were scaled to the unmethylated, unstimulated control. The results are the average and standard error from three separate experiments of eight replicates each. * = p<0.0001 compared with control. (D) The minimal *Ins2* promoter construct was treated and analyzed as in 1C. There was no significant effect of methylation or stimulation on minimal promoter activity (one-way ANOVA p = 0.9928). (E) The mouse *Ins2* promoter construct was methylated individually at each CpG site (white bars) or mock-methylated (black bar) and then transfected and analyzed as in 1B. The results are the average and standard error from three to five experiments of eight replicates each. * = p<0.0001 compared with control.

Examination of the mouse *Ins2* promoter revealed three CpG dinucleotide sequences located at positions −414, −182, and −171 bp relative to the transcription start site (Figs. 1A and 2A). To test whether these CpG sites have a specific methylation pattern in pancreatic beta cells, we compared the insulin promoter CpG methylation pattern in pancreatic beta cells with various other tissues and with the NIT-1 mouse insulinoma cell line. A portion of the isolated beta cells were immunostained with insulin and glucagon antibodies which showed that more than 70% of the cells were positive for insulin (data not shown). Genomic DNA samples from various mouse tissues, including pancreatic islets, were bisulfite-treated and analyzed by methylation-specific PCR (Table 1) followed by sequencing. The sequencing data revealed that the three CpG sites in the *Ins2* promoter are uniquely unmethylated in pancreatic beta cells and in the insulin-producing NIT-1 beta cell line [18] but predominantly methylated in other tissues (Fig. 1A).

**Table 1:** Primers for bisulfite sequencing, reporter constructs, and ChIP assays.

Primers	Position	Strand	Sequence (5′ to 3′)
*Ins2* bisulfite	−516– −126	Sense	TTTAAGTGGGATATGGAAAGAGAGATA
		Antisense	ACTACAATTTCCAAACACTTCCCTAATA
*Ins2* reporter	−480– +15	Sense	GAGCTCGGACCATTAAGTGCCTTGCTGCCT
		Antisense	AAGCTTGGATCACTTAGGGCTGGTGGTTACTGG
*Ins2* minimal	−169– +15	Sense	GAGCTCCTTTCTGCAGACCTAGCACCAGG
		Antisense	AAGCTTGGATCACTTAGGGCTGGTGGTTACTGG
Plasmid ChIP	*Ins2* −190– luc2 +21	Sense	GGTGTTGACGTCCAATGAGCGCTTTCTGCAGA
		Antisense	CGCTGGGCCCTTCTTAATGTTTTTGGCATCTTCCA
Genomic ChIP	−276– −78	Sense	CTGGAACAATGTCCCCTGCTGTGAACTGGTTC
		Antisense	TTAAGGGCTCCAGGTGGGGTAGGTCAGCAGA
*INS* bisulfite	−385– +31	Sense	TTTGGGGATAGGGGTTTGGGGATAGTA
		Antisense	CCTCTTCTAATACAACCTATCCTAAAAAACTAAAAACTAC
*INS* reporter	−385– +24	Sense	GAGCTCTGTGGGGACAGGGGTCTGGGGA
		Antisense	AAGCTTCTGATGCAGCCTGTCCTGGAGGGCTGAGG

There are nine CpG sequences in the human insulin promoter (−385 to 0 bp) located at positions −357, −345, −234, −206, −180, −135, −102, −69 and −19 bp relative to the transcription start site (Fig. 1B). To map the tissue-specific methylation pattern of the human insulin gene, purified human islets were dissociated and the beta cell fraction was separated from non-beta cells using Newport Green FACS sorting [19]. The enriched beta cell fraction was 70% insulin positive (data not shown) by immunostaining. We compared the methylation pattern of the human *INS* gene promoter in the beta cell fraction with pancreatic tissue leftover from islet isolation, which is mostly pancreatic acinar cells and contains less than 1% beta cells [20]. As shown in Figure 1B, the insulin promoter in the insulin-producing human beta cell fraction is unmethylated, but it is predominantly methylated in the beta cell depleted fraction.

These results demonstrate that the adult human and mouse insulin gene promoters exhibit a tissue-specific pattern of DNA methylation.

### Methylation suppresses insulin gene expression

Next, we tested whether methylation affects expression from the insulin gene promoter. A reporter gene construct was made by inserting the human *INS* promoter into a luciferase expression plasmid (pGL4.10INS421bp; Fig. 2A). Additionally, two mouse *Ins2* luciferase reporter constructs were made, one (pGL4.10*Ins2*500bp) using *Ins2* promoter sequences from −480 to +15 bp containing all three CpG sites, and the other (pGL4.10*Ins2*MP) using *Ins2* promoter sequences from −169 to +15 bp which does not contain any CpG sites (Fig. 2A). Each promoter fragment was mock-methylated or methylated with M.SssI CpG methyltransferase prior to insertion into the pGL4.10 luciferase expression plasmid (so that only the promoter fragments were methylated) and then used to transfect mouse NIT-1 cells, which exhibit both basal and stimulated insulin gene expression. As shown in Figure 2B, methylation of the human *INS* promoter suppressed reporter gene expression in NIT-1 cells about eighty-five percent (1.00±0.13 vs. 0.150±0.02 RLU; p<0.001). Similarly, CpG methylation significantly suppressed expression from the mouse *Ins2* promoter-driven luciferase reporter construct (Fig. 2C; 1.00±0.10 vs. .212±0.15 RLU; p<0.001). By contrast, the minimal Ins2 promoter, pGL4.10Ins2MP, which lacks the CpG sites, was not affected by methyltransferase treatment (Fig. 2D; 1.00±0.31 vs. 0.99±0.19 RLU; p = 0.911), although the total activity of the minimal promoter was approximately thirty-fold lower than the full-length promoter.

Glucose is the major nutrient regulator of pancreatic beta cell function and coordinately regulates insulin gene transcription, with multiple elements of the promoter involved in glucose responsiveness [21], [22]. The A3, E1, and C1 regions are the major glucose-responsive transcription control elements of the insulin gene and these elements are conserved in the *Ins2* promoter region [23], [24]. Of the three CpG sites, the second CpG site at −182 bp of insulin promoter is part of the cyclic adenosine monophosphate (cAMP) responsive element (CRE), which is conserved through many species and is required for maximal insulin gene expression [16]. Forskolin increases intracellular cAMP levels in the beta cell and activates kinases that phosphorylate CRE binding proteins which stimulate CRE-dependent transcription [25]. We therefore tested the effects of a combination of high glucose and forskolin stimulation in reporter assays using methylated or mock-methylated pGL4.10*Ins2*500bp. Glucose and forskolin treatment significantly stimulated the mock-methylated reporter (Fig. 2C black bars; 1.00±0.10 vs. 1.65±0.10, p<0.0001), however did not stimulate the methylated reporter (Fig. 2C white bars; 0.21±0.04 vs. 0.35±0.15, p = 0.385), nor the minimal *Ins2* promoter, pGL4.10*Ins2*MP (Fig. 2D black bars; 1.00±0.31 vs. 1.01±0.16, p = 0.986). These results indicate that CpG sites and their demethylation status are important for both basal and stimulated insulin gene expression.

To assess the importance of the individual methylation events on insulin gene expression, reporter plasmids were made in which each CpG site was methylated individually, and then tested for the effects on gene expression. Of the three sites, only methylation of the CpG site within the CRE (−182) was able to independently suppress insulin promoter activity, and only by about 50% (Fig. 2E; 1.00±0.08 vs. 0.56±0.05, p<0.0001). Methylation of the proximal site (−171) had no effect on gene expression, while methylation of the distal site (−414) paradoxically increased expression from the *Ins2* promoter by almost two-fold (Fig. 2E; 1.00±0.08 vs. 1.86±0.15, p<0.0001). This shows that methylation-dependent suppression of the insulin promoter is not simply additive, and that other mechanisms are likely present in the cell that cooperate with DNA methylation to turn off the insulin gene.

### Methylation indirectly inhibits factor binding to the CRE

**Figure 3:**
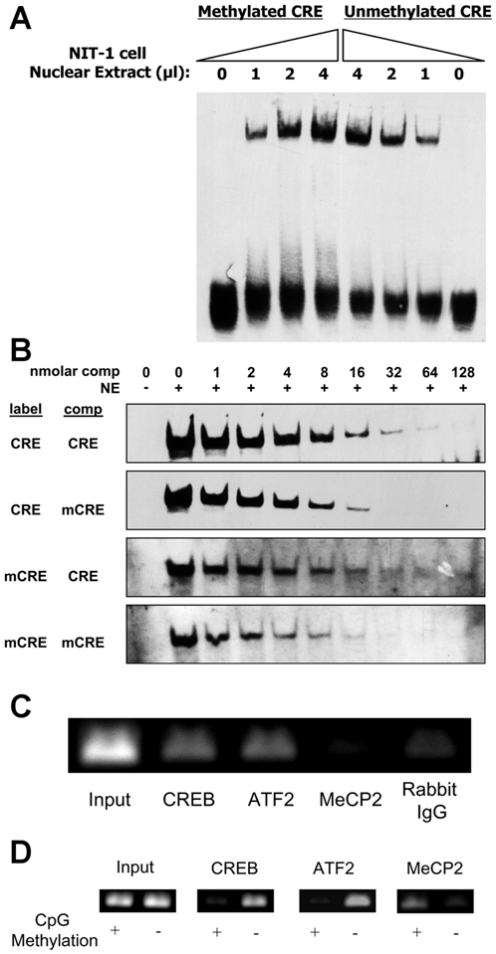
Effect of methylation on transcription factor binding to the *Ins2* promoter. (A) Increasing amounts of NIT-1 nuclear extract was bound to methylated or unmethylated mouse *Ins2* promoter CRE oligonucleotide in an Electrophoretic Mobility Shift Assay (EMSA). There was a dose-dependent increase in binding to both the methylated and unmethylated CRE with increasing amounts of NIT-1 cell nuclear extract. (B) The binding of NIT-1 nuclear proteins to one nanomolar of either labeled methylated (mCRE) or unmethylated (CRE) was competed with increasing concentrations of unlabeled CRE or mCRE. The figure is one representative of four experiments. (C) Chromatin immunoprecipitation (ChIP) analysis of the endogenous mouse *Ins2* gene promoter in NIT-1 cells. Genomic DNA was immunoprecipitated with anti-CREB, anti-ATF2, anti-MECP2, and control IgG. The results indicate that CREB and ATF2, but not MeCP2, are normally associated with the endogenous insulin promoter. (D) Methylated or mock-methylated pGL4.10*Ins2*500bp (Fig. 2A) was transfected into NIT-1 cells and then ChIP was carried out with anti-CREB, anti-ATF2, and anti-MeCP2 antibodies. Methylation of the *Ins2* promoter decreases the binding of CREB and ATF2, but increases the binding of MeCP2.

Suppression of gene expression by methylation of the CpG at −182 bp suggested that methylation may regulate the binding of transcription factors to the CRE site. CREB and ATF2 are reported to bind to the *Ins2* CRE site and play an important role in insulin transcription [26]. Several studies have demonstrated that CpG methylation of CRE sites suppresses the binding of transcription factors and represses gene expression [27]–[29]. To test the effect of methylation on factors binding to the CRE site, double stranded 24 bp oligomers of the *Ins2* promoter CRE site were synthesized and methylated *in vitro* or mock methylated. Methylation of the oligonucleotides was confirmed by inhibition of AatII restriction digest (not shown). Electrophoretic mobility shift assays (EMSA) were carried out using NIT-1 nuclear extracts to test whether methylation inhibited factor binding to the CRE site. In contrast to other studies [27], [28], we found dose-dependent binding to the CRE site regardless of the methylation status (Fig. 3A). To determine if the factors binding to methylated and unmethylated oligonucleotide were able to be displaced, increasing concentrations of unlabeled oligonucleotide were allowed to compete with the labeled oligonucleotides for binding (Fig. 3B). Both methylated and unmethylated unlabeled oligonucleotides effectively competed with the labeled probes for factor binding, indicating that methylation had no effect on factor binding to the *Ins2* CRE *in vitro*. According to this result, the methylation-dependent repression of insulin gene expression was not due to direct inhibition of transcription factor binding.

Alternatively, another protein(s) might bind exclusively to the methylated CRE site and regulate *Ins2* transcription. The methyl-CpG-binding protein MeCP2 has been reported to bind to methylated CpG motifs [30] in CRE sites, which may block binding of other factors. Additionally, the binding of MeCP2 to methylated DNA sites in gene promoters recruits histone deacetylases, causing deacetylation of histone protein tails and chromatin condensation around the gene promoter, resulting in transcriptional repression [31]. We used chromatin immunoprecipitation (ChIP) experiments to determine the factors binding the CRE in the endogenous *Ins2* gene in NIT-1 insulinoma cells. As shown in Figure 1A, this promoter is completely demethylated, and both CREB and ATF2, but not MeCP2, were associated with the endogenous gene as previously reported (Figure 3C) [26].

To investigate the effect of methylation on factor binding *in vivo*, ChIP experiments were carried out using methylated or mock methylated reporter plasmids transfected into NIT-1 cells. Whole pGL4.10*Ins2*500bp plasmid was methylated using M.SssI CpG methyltransferase and methylation status was confirmed by digestion with the methylation-sensitive enzyme HpaII and methylation-insensitive enzyme MspI, and it was also confirmed that DNA methylation did not alter PCR efficiency (data not shown). To avoid background from the endogenous gene, ChIP PCR primers were designed to amplify only the transfected reporter DNA (Fig. 2A). NIT-1 cells were transfected with whole methylated or unmethylated pGL4.10*Ins2*500bp, cross-linked with formaldehyde, and precipitated with specific antibodies against CREB, ATF2, and MeCP2. The ChIP data shows that CpG methylation inhibited the binding of CREB and ATF2 to the *Ins2* promoter CRE site, but increased the binding of MeCP2 (Fig. 3D). Therefore, the binding of the CRE-binding proteins in NIT-1 cells to the insulin promoter is inhibited by methylation inside the cells, but not *in vitro*. This implies that MeCP2, and possibly other methylated CpG binding proteins, are binding to the methylated promoter and preventing binding of the activators, resulting in a decrease in insulin gene expression. Similar reciprocal binding of CREB and MeCP2 has also been reported by another group [28].

### Insulin gene is demethylated during differentiation of insulin-producing cells

Induction of insulin gene transcription occurs rapidly (<30 min) following glucose stimulation and involves acute changes in chromatin structure [32]. However, in the present study the insulin promoter was demethylated in adult insulin-producing cells even when cells were cultured at normal glucose levels (7 mM for mouse islets, 5.5 mM for human islets; Fig. 1), implying that methylation may not be important for acute regulation of insulin gene expression. Therefore we reasoned that beta cell-specific demethylation may be a developmentally regulated process involved in the differentiation of beta cells. This would mean that either the insulin gene is demethylated in the embryo and becomes preferentially methylated in all cells but the beta cells, or the reverse is true, i.e. the gene is methylated in the embryo and becomes specifically demethylated in beta cells. To evaluate these possibilities, we followed the changes in the methylation pattern as mouse embryonic stem cells differentiated into insulin-expressing cells.

Many groups are trying to generate insulin-producing beta cells from several types of adult and embryonic cells, however, the conversion ratio to insulin positivity is very low [20], [33]. Our prior studies had succeeded in enriching for insulin-positive cell clusters derived from a mouse embryonic stem cell (mESC) line [34] that has one allele of the neurogenin 3 (Ngn3) gene replaced with Enhanced Green Fluorescent Protein (EGFP) reporter [35]. During development, Ngn3 marks the proendocrine cells of the developing pancreas and is required for their differentiation [36], [37], so this method enriches for cells which are already proceeding down a pathway to hormone expression. In this method, mESC were allowed to differentiate into embryoid bodies (EB) for six days in culture, and then the EB were collected, washed, and cultured in tertiary media for 9 to 11 days. At the end of tertiary culture, the cells were dissociated and FACS-sorted into GFP positive (Ngn3-EGFP^+^) and negative (Ngn3-EGFP^−^) populations, representing proendocrine and non-endocrine fractions, respectively. Since the half-life of EGFP transcript is longer than Ngn3, some of the EGFP+ cells may already proceed down a pathway to hormone expression. The Ngn3-EGFP^+^ population was induced to further differentiate by 3-dimensional culture in extracellular matrix, and colonies of differentiated insulin-expressing cells were handpicked for analysis. Genomic DNA was isolated from cells at each of the six stages and also from mouse liver as a negative control and mouse beta cells and NIT-1 cells as positive controls. The DNA methylation pattern of the insulin promoter in each of these cell populations was determined by pyrosequencing.

**Figure 4:**
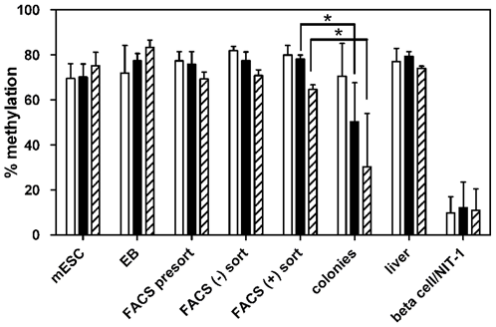
Demethylation of the *Ins2* promoter during differentiation of mouse embryonic stem cells to insulin-expressing colonies. Mouse embryonic stem cells from Ngn3-EGFP mice were induced to differentiate through several stages into insulin-expressing colonies as previously described [34]. Genomic DNA from each stage was isolated, bisulfite-treated, and the methylation status was determined by pyrosequencing. The methylation status of the three CpG sites is plotted individually: −171 (white), −182 (black), −414 (hatched). The bars represent the averages and standard deviations of three independent stem cell experiments. Genomic DNA samples from mouse liver and purified beta cells were processed in parallel with each experiment as negative and positive controls, respectively. * p<0.05 compared with previous stage.

As shown in Figure 4, the insulin gene promoter in mESC is methylated on all three CpG sites, similar to the liver DNA control. The methylation status is maintained through embryoid body formation (Fig. 4; EB) and endodermal differentiation in the tertiary culture (Fig. 4; FACS presort). Surprisingly, the insulin promoter in the Ngn3-EGFP^+^ proendocrine cell population (Fig. 4.; FACS (+) sort), which exhibits detectable *Ins2* gene expression [34], was still fully methylated. It was not until the final stage of *in vitro* differentiation (Fig. 4; colonies) that there was significant demethylation of two (−182 and −414) of the three CpG sites. This result shows that complete demethylation of the insulin promoter is a late step in the differentiation of the beta cell phenotype, and is preceded by low level insulin gene expression. It should be noted that the level of insulin gene expression, even in the final stage of *in vitro* differentiation, is substantially lower than that of fully differentiated mouse beta cells.

## Discussion

The insulin molecule is central to metabolic homeostasis, and as such insulin activity is regulated at multiple levels. Previous studies have established the regulation of insulin activity at the transcriptional [21], [22], [24], post-transcriptional [32], translational [38], [39], secretory [40], and even post-receptor levels [41], [42]. In this study we have demonstrated that human insulin gene and mouse insulin2 gene expression are also controlled epigenetically by methylation of specific CpG sites in the promoter regions. In non-insulin expressing cells, some or all of these sites are methylated, whereas in insulin-expressing cells they are completely demethylated.

In general, DNA methylation has a suppressive effect on gene transcription. We show that this is also true of the human and mouse insulin promoters, where complete methylation suppressed reporter gene expression by 85% and 79%, respectively (Figs. 2B and 2C). However, methylation of the individual sites in the mouse promoter revealed only one site, the CpG at position −182, which significantly suppressed gene expression, and only by about 50%. Paradoxically, methylation of the distal CpG at position −414 significantly increased gene expression by 86%. Since complete methylation suppresses insulin promoter activity, these observations imply that methylation-dependent control of insulin gene expression is indirect, probably involving a higher levels of DNA structure such as nucleotide positioning and chromatin compaction.

The CpG site at position −182 in the mouse Ins2 promoter is located within a cyclic-adenosine monophosphate response element (CRE) which was previously shown to be involved in insulin gene expression. Previous studies have demonstrated that CRE methylation suppresses transcription of other genes, usually by direct inhibition of binding of the transcription factor CREB. Therefore, we investigated the effect of methylation on binding of beta cell factors to the CRE sequence in the mouse *Ins2* gene. In agreement with other groups, we found that the transcription factor ATF2 binds to the insulin promoter CRE and that overexpression of ATF2 increases insulin gene expression, whereas CREB overexpression actually suppresses the insulin promoter (data not shown). We also found that binding of both ATF2 and CREB to the insulin promoter CRE *in vivo* was inhibited by methylation. However, in contrast to other gene promoters, we found that methylation did not directly inhibit transcription factor binding to the CRE in the insulin promoter. Importantly, methylation increased the binding of the methylated DNA binding protein MeCP2. We believe that the binding of MeCP2 to the methylated CpG in the CRE site may compete with binding of other factors, thus contributing to the suppression of gene expression.

Yet the predominant suppressive effect of binding of MeCP2 to the insulin gene is probably not due to blocking of transcriptional activators since methylation of the CRE CpG alone results in only 50% inhibition, but rather MeCP2 binding likely recruits histone deacetylases that maintain the chromatin in a compacted state. Wu and Gilbert [43] showed long ago that the promoter of the rat insulin 2 gene exhibits tissue-specific sensitivity to DNase I, indicating a relaxed chromatin structure in this region in insulin-producing cells. More recently the Mirmira laboratory has provided a mechanism for this observation, showing that there is a beta cell-specific shift in nucleosome positioning in the insulin promoter, allowing the binding of the PDX-1 transcription factor [44], which recruits histone methylase to the promoter [45], thus stabilizing the open chromatin structure and providing the opportunity for transcription. We speculate that an initiating event in this process is the beta cell-specific demethylation of the insulin locus resulting in the relaxation of the chromatin structure and repositioning of the nucleosomes.

Therefore, unlike other mechanisms controlling insulin activity, methylation is likely involved in the development of insulin-producing cells rather than in daily metabolic regulation. When one particular developmental model was examined, i.e. the transition of mouse embryonic stem cells to endodermal-like cells and then into an early pancreatic lineage *in vitro*, it appeared that there is a transition in the insulin promoter from full methylation to partial demethylation. However, decrease in methylation at specific sites occurs very late in the differentiation program and was incomplete in this model, even in the isolated Ngn3-EGFP^+^ pancreatic endocrine lineage cells. This indicates that demethylation is one of the very late steps in differentiation of insulin-producing cells. This may be one of the reasons that no one has yet been able to achieve efficient differentiation of functional insulin-producing cells *in vitro*. As yet, the precise mechanism of gene-specific DNA demethylation has not been unraveled. It may be a passive process involving replication in absence of maintenance DNA methylation. Alternatively, recent studies indicate that active DNA demethylation may occur and this pathway may involve interaction between cytosine deaminase and thymine-DNA glycosylase [46], [47]. The developmental control of gene-specific DNA demethylation is the subject of ongoing investigations.

In conclusion, this study demonstrates that DNA methylation suppresses insulin gene expression and that the insulin gene promoter is specifically demethylated in insulin-producing cells. Methylation recruits MeCP2, and possibly other methylated DNA binding proteins, resulting in decreased binding of the transcriptional activators, ATF2 and CREB. Furthermore, demethylation of the insulin gene appears to be an important and potentially rate-limiting step in the differentiation of insulin-producing cells from embryonic stem cells.

## Materials and Methods

### Ethics Statement

Animals used in this study received high quality animal care consistent with the Public Health Service Policy on Humane Care and Use of Laboratory Animals, the Guide for the Care and Use of Laboratory Animals, and the Animal Welfare Act. The animal care facility at the City of Hope has been approved by the National Institutes of Health, registered with the U.S. Department of Agriculture, and fully accredited by the Association for Assessment and Accreditation of Laboratory Animal Care International (AAALAC). The experiments were designed to utilize the minimum number of animals required to obtain valid results.

### Mouse islet cell preparations

Pancreata from NOD*scid* or Balb/c mice were digested with 1 mg/ml Liberase (Sigma-Aldrich, St. Louis, MO) for 11 minutes in 37°C and then purified islets were separated using Histopaque-1077 (Sigma-Aldrich, St. Louis, MO) followed by hand picking. Isolated islets were cultured overnight and dissociated using Accutase (Innovative Cell Technologies, CA) for 8 minutes and one tenth of the dissociated cells were used for immunocytochemistry to determine purity and composition.

### Human pancreatic beta cell sorting

Human islet cell isolation at the City of Hope National Medical Center was performed with the approval of the Institutional Review Board (IRB# 01083 and 05058). Written informed consent from the donors for research use of tissue in this study was obtained prior to acquisition of the organ. Ten thousand Islet Equivalent (IEQ) human islets from four different donors in independent experiments were incubated with TrypLE (Invitrogen, Carlsbad, CA) at 37°C for 15 min. Dissociated islet cells were passed through the 100 and 40 micron strainers. The cells were incubated with 1microM Newport Green (Invitrogen, Carlsbad, CA) at 37°C for 1 hour. The cells were resuspended with phosphate buffer containing 0.1% human serum albumin and 2 mM MgCl_2_. MoFlo High-Performance Cell Sorter (DAKO, Carpinteria, CA, USA) was used for Newport Green positive cell sorting. One tenth of the sorted cells were used for immunocytochemistry and genomic DNA was isolated from the remaining cells. Human non-islet pancreatic tissue containing less than 0.1% islet cells was obtained following the islet isolation procedure by the Southern California-Islet Cell Resource center at the City of Hope.

### Immunocytochemistry

Immunocytochemical analyses were performed on cells attached to polylysine-coated coverslips and fixed with 4% paraformaldehyde, cold methanol and acetone. The following primary antibodies were used for immunocytochemical staining: guinea pig anti-insulin (Millipore, St Charles, MO), rabbit anti-glucagon (Millipore, St Charles, MO). Corresponding secondary antibodies against each first antibody/immunoglobulin were either conjugated with fluorescein isothiocyanate (FITC) or Texas red (Jackson Immuno-Research Laboratories, West Grove, PA). Stained samples were embedded in Vectashield (Vector Laboratories, Burlingame, CA) and examined under an Olympus BX51 fluorescent microscope, equipped with a Pixera 600 CL cooled CCD camera (Olympus America, Melville, NY). A picture for each individual color was separately captured using Pixera Viewfinder 3.0 acquisition software (Pixera, Los Gatos, CA). Cell percentages were determined by manual counting of at least six randomly chosen fields.

### Cell culture

Mouse beta-cell line NIT-1 cells [18] were purchased from American Type Culture Collection (Manassas, VA, USA) and were cultured in Hams F12K media supplemented with 10% fetal bovine serum and antibiotics. Cells were maintained at 37°C in a humidified atmosphere containing 95% air and 5% CO2. Cells were passaged by trypsinization and subcultured on a weekly basis. Passage 2 or 3 cells were used for all the experiments.

### Isolation of genomic DNA

Genomic DNA was obtained from mice liver, spleen, kidney, brain, muscle, blood, isolated pancreatic islet of NOD*scid* mice or Balb/c mice using ZR genomic DNA kit (Zymo Research, Orange, CA). Tissues were obtained from four NOD*scid* mice and three Balb/c mice for comparing tissue-specific methylation patterns. We observed no strain-specific differences in methylation patterns. TRIzol reagent (Molecular Research Center, Cincinnati, OH, USA) was used for human islet genomic DNA preparation.

### Bisulfite genomic sequencing

Nucleotide sequences of the mouse *Ins2* gene (GeneID: 16334) and the human *INS* (GeneID: 3630) gene were obtained from Genbank and the potential methylation sites (i.e. CG dinucleotides) were identified. Genomic DNA samples were treated with EZ DNA methylation kit (Zymo Research, Orange, CA) according to the manufacturer's recommendation. The mouse *Ins2* gene or human insulin gene was then amplified with pairs of gene-specific primers (Table 1) in a mixture containing 100 ng bisulfite-modified DNA. PCR was done using Hot Star-*Taq* polymerase (Qiagen, Valencia, CA). Each PCR fragment was TA cloned into pCR2.1-TOPO vector (Invitrogen, Carlsbad, CA) and transformed with the TOP10-competent cells (Invitrogen, Carlsbad, CA). 5–24 clones of each sample were selected and sequenced with M13F primer or M13R primer. Sequencing was performed by the DNA Sequencing Lab in the Beckman Institute at the City of Hope. There were no discernible strain-specific differences in the tissue-specific methylation patterns between the NOD*scid* and Balb/c mice.

### Construction of reporter gene plasmids

pGL4.10-Basic vector (Promega, Madison, WI) was digested with SacI/HindIII and treated with alkaline phosphatase (CIP) (New England BioLabs, Ipswich, MA). Fragments of the mouse *Ins2* promoter (−480– +15 bp containing 3 CpG sites (pGL4.10*Ins2*500bp) and 169– +15 bp containing no CpG sites (pGL4.10*Ins2*MP) and of the human *INS* promoter (−385– +24 bp containing nine CpG sites (pGL4.10*INS*421bp) were amplified by PCR using genomic DNA and primers that added a 3′ SacI site and a 5′ HindIII site (Table 1). PCR products were inserted into pCR2.1 TOPO vector using TA cloning kit and amplified in ZYM-505 medium [48]. The plasmid sequences were confirmed by DNA sequencing. The cloned promoter fragments were excised and subcloned upstream of the firefly luciferase gene in the pGL4.10-Basic vector (Promega, Madison, WI) using Fastlink ligation kit (Epicentre, Madison, WI, USA) according to the manufacturer's recommendation and transformed into TOP10 competent cells (Invitrogen, Carlsbad, California) for plasmid production.

### In vitro methylation and mock methylation of insulin2 or human insulin promoter

pCR2.1 plasmids containing the promoter region of *Ins2* SacI–480 to +15 bp-HindIII, SacI–169– +15 bp-HindIII, and the promoter of human insulin SacI–385 to +24 bp-HindIII were digested with SacI/HindIII and insert DNA were gel purified and methylated using M.*SssI* CpG methyltransferase (New England Biolabs, Ipswich, MA), which methylates all cytosine residues within the double-stranded dinucleotide recognition sequence 5′-CG-3′. One microgram promoter DNA was methylated using 10 U of M.*Sss*I CpG methyltransferase, or in a parallel control reaction, mock-methylated in the absence of enzyme. Methylated and mock-methylated plasmids were religated into SacI/HindIII-restricted pGL4.10-Basic vector [49]. The correctly ligated promoter constructs were identified and gel purified, quantitated by spectrophotometry, and confirmed by restriction enzyme digestion prior to transfection. For the *Ins2* promoter, the CpG methylated insert was confirmed by digestion with the methylation-sensitive restriction enzyme AatII, which recognizes the GACGTC site at −184–179 bp of *Ins2* promoter. For the *INS* promoter, the CpG methylated insert was confirmed with HpaII and MspI (New England Biolabs, Ipswich, MA). HpaII is a methylation-sensitive restriction endonuclease that cleaves DNA at CCGG sequences when the internal cytosine residue is unmethylated on both strands. MspI cleaves DNA regardless of methylation status of the internal cytosine and serves as a control for digestion efficiency.

### Site specific methylation of the *Ins2* promoter

There are 3 CpG sites in the *Ins2* proximal promoter at positions −414, −182, and −171 bp. Site specific methylation of pGL4.10*Ins2*500bp was carried out with the QuikChange Site Directed Mutagenesis Kit using a protocol adapted from Martinowich et al. [50]. Briefly, methylated primers from sequences around CpG sites −414, −182, and −171 bp of the *Ins2* promoter were purchased from IDT (Coralville, IA). The following primers and their reverse complements, containing methylated CpG, were used: −414 bp CpG: 5′-GAGAGAGAGCTGGGGACTCGGCTGAGTTAAGA-3′; −182 and −171 bp CpGs: 5′-CTAAGTAGAGGTGTTGACGTCCAAATGAGCGCTTTCTGCAGAC-3′. Control unmethylated vector was generated using the unmethylated primers for the −182 and −171 bp sequences. PCR was carried out according to the manufacturer's protocol with 100 ng of pGL4.10*Ins2*500bp per reaction. Following the PCR, the reaction was treated with DpnI for 2 h to remove the template DNA. The product was purified using DNA clean and concentrater-5 (Zymo Research, Orange, CA). DNA concentration was quantitated by gel electrophoresis.

### Transient transfection and luciferase reporter assays

The unmethylated (mock-methylated), totally methylated, or point methylated *Ins2* promoter/firefly luciferase fusion genes, pGL4.10*Ins2*500bp or pGL4.10*Ins2*MP were transfected in NIT-1 cells using Fugene 6 (Roche, Indianapolis, IN, USA). In each experiment, the phRL-TK, encoding *Renilla* luciferase (Promega, Madison, WI), was cotransfected as normalization control. Luminescence was measured 48 h after transfection using the dual-luciferase reporter assay system (Promega, Madison, WI). For stimulation assays, four hours before the assay the medium was replaced with 100 µl RPMI and 10% fetal bovine serum with 2.8 mM glucose or 16.7 mM glucose with 10 µM of forskolin. Reporter activity was normalized by calculating the ratio of *firefly* to *Renilla* values. Each construct was tested in three to five independent transfections.

### Electrophoretic Mobility Shift Assays

Sense and antisense strand oligonucleotides corresponding to positions −194 to −160 of the mouse Ins2 promoter and containing CRE and two CpG sites (sense: 5′-tagaggtgttgaCGtccaatgagCGctttctgcag-3′; antisense: 5′-ctgcagaaagCGctcattggaCGtcaacacctcta-3′; CpG sites are capitalized, the CRE is underlined) were synthesized by IDT. The oligonucleotides were labeled with biotin at the 3′-end using the Pierce Biotin 3′ End DNA Labeling Kit (Rockford, IL, USA), then annealed, and an aliquot was methylated using M.SssI CpG methylase (New England Biolabs, Ipswich, MA). The methylation status of the oligonucleotide was confirmed by CpG methylation sensitive restriction digest with AatII. Nuclear extracts of NIT-1 cells were prepared according to Schreiber et al [51] with the addition of 1 mM NaF and 0.2 mM NaVO_4_, and the protein concentration determined using the BioRad Protein Assay (Hercules, CA, USA). Methylated or unmethylated oligonucleotides (20 fmol) were mixed with 2–8 µg NIT-1 nuclear protein in 20 µl of 15 mM Tris-HCl, 50 mM KCl, 0.1 mM EDTA, 1 mM DTT, 0.1 mg/ml BSA, 10% glycerol, 0.05% NP40, 0.5 µM biotin, and 0.1 mg/ml poly-(dIdC) and incubated for 30 minutes at room temperature. Binding was assessed by separation on 6% polyacrylamide/0.5x TBE gels, transfer to nylon membranes, and detection with LightShift Chemiluminescent EMSA reagents (Pierce, Rockford, IL, USA).

### Transfected plasmid ChIP

pGL4.10*Ins2*500bp (40 µg) was treated with M.SssI (60 U) overnight in 37°C. The unmethylated and methylated plasmids (200 ng) were digested with 20 U of either HpaII or MspI at 37°C overnight to confirm the methylation status. NIT-1 cells growing on 3×10-cm culture dishes were transfected with 18 µg of whole CpG methylated or unmethylated pGL4.10*Ins2*500bp using Fugene6 transfection reagent. Chromatin immunoprecipitation (ChIP) was carried out using enzymatic shearing method kit (Active Motif, Carlsbad, CA, USA) according to the manufacturer's recommendations with the following antibodies: mouse anti-CREB (Invitrogen, Carlsbad, CA), rabbit polyclonal anti-ATF2 (Santa-Cruz, Santa Cruz, CA, USA), rabbit polyclonal anti-MeCP2 (Upstate, Chicago, IL, USA). Amplification of the immunoprecipitated DNA was achieved using Hot Star *Taq* DNA polymerase and 5 µl of either immunoprecipitated DNA, or a 1∶10 dilution of input chromatin. Experimental reactions were performed to determine optimal PCR conditions so that the yield of PCR products was dependent on the amount of input DNA (data not shown). The conditions for all reactions were as follows: 94°C for 15 min, followed by 32 cycles at 94°C for 30 seconds, 61°C for 30 seconds, 72°C for 1 min, and 72°C for 10 min. Primers were designed to amplify only reporter construct DNA (Fig. 2A and Table 1). PCR products were electrophoresed on 2% agarose gels and stained with ethidium bromide.

### Genomic ChIP

NIT-1 cells growing on 3×10-cm culture dishes were used for genomic ChIP. ChIP was carried out with the same antibodies utilized in the plasmid ChIP. The normal control rabbit IgG (Alpha Diagnostic, Carlsbad, CA, USA) was used for the negative control. The conditions for all reactions were as follows: 94°C for 15 min, followed by 33 cycles at 94°C for 30 seconds, 63°C for 30 seconds, 72°C for 1 min, and 72°C for 10 min.

### Pyrosequencing

Mouse embryonic stem cell were differentiated and sorted and cultured as previously described [34], and genomic DNA from samples at each stage were isolated and bisulfite converted. Pyrosequencing of the *Ins2* promoter region was done by EpigenDX (Worcester, MA) using 5′-biotinylated primer sets. Liver and beta cell bisulfite treated genomic DNA PCR were analyzed simultaneously as negative and positive controls, respectively. The percentages of methylation at each CpG site in the Ins2 promoter are shown for each stage of differentiation.
