# Correction: Insulin Gene Expression Is Regulated by DNA Methylation

**DOI:** 10.1371/annotation/947a8d4a-3585-4b23-ac84-b47a255a70d9

**Published:** 2009-10-09

**Authors:** Akio Kuroda, Tibor A. Rauch, Ivan Todorov, Hsun Teresa Ku, Ismail H. Al-Abdullah, Fouad Kandeel, Yoko Mullen, Gerd P. Pfeifer, Kevin Ferreri

The following information was missing from the Funding section: Additional funding was provided by NIH grant 5R01DK081587-01. 

